# Biofilm Formation and Antimicrobial Susceptibility of *E. coli* Associated With Colibacillosis Outbreaks in Broiler Chickens From Saskatchewan

**DOI:** 10.3389/fmicb.2022.841516

**Published:** 2022-06-17

**Authors:** Murugesan Sivaranjani, Madeline C. McCarthy, Michelle K. Sniatynski, Linzhi Wu, Jo-Anne R. Dillon, Joseph E. Rubin, Aaron P. White

**Affiliations:** ^1^Vaccine and Infectious Disease Organization, Saskatoon, SK, Canada; ^2^Department of Biochemistry, Microbiology and Immunology, University of Saskatchewan, Saskatoon, SK, Canada; ^3^Department of Veterinary Microbiology, University of Saskatchewan, Saskatoon, SK, Canada

**Keywords:** antibiotics, antimicrobial resistance (AMR), Avian pathogenic *E. coli* (APEC), biofilm, disinfectant, colibacillosis, poultry

## Abstract

The global poultry industry has grown to the extent that the number of chickens now well exceeds the number of humans on Earth. *Escherichia coli* infections in poultry cause significant morbidity and economic losses for producers each year. We obtained 94 *E. coli* isolates from 12 colibacillosis outbreaks on Saskatchewan farms and screened them for antimicrobial resistance and biofilm formation. Fifty-six isolates were from broilers with confirmed colibacillosis, and 38 isolates were from healthy broilers in the same flocks (cecal *E. coli*). Resistance to penicillins, tetracyclines, and aminoglycosides was common in isolates from all 12 outbreaks, while cephalosporin resistance varied by outbreak. Most *E. coli* were able to form biofilms in at least one of three growth media (1/2 TSB, M63, and BHI broth). There was an overall trend that disease-causing *E. coli* had more antibiotic resistance and were more likely to form biofilms in nutrient-rich media (BHI) as compared to cecal strains. However, on an individual strain basis, there was no correlation between antimicrobial resistance and biofilm formation. The 21 strongest biofilm forming strains consisted of both disease-causing and cecal isolates that were either drug resistant or susceptible. Draft whole genome sequencing indicated that many known antimicrobial resistance genes were present on plasmids, with disease-causing *E. coli* having more plasmids on average than their cecal counterparts. We tested four common disinfectants for their ability to kill 12 of the best biofilm forming strains. All disinfectants killed single cells effectively, but biofilm cells were more resistant, although the difference was less pronounced for the disinfectants that have multiple modes of action. Our results indicate that there is significant diversity and complexity in *E. coli* poultry isolates, with different lifestyle pressures affecting disease-causing and cecal isolates.

## Introduction

Colibacillosis is a broad term for infections caused by pathogenic strains of *Escherichia coli*, and it is known to affect wide range of farm animals including poultry, pigs, and calves (Foster and Smith, 2009; Guabiraba and Schouler, 2015; Renzhammer et al., 2020). Avian colibacillosis is an infectious disease of poultry caused by Avian pathogenic *E. coli* (APEC) (Dziva and Stevens, 2008; Kemmett et al., 2013; Guabiraba and Schouler, 2015). It is associated with collection of extraintestinal infections including acute fatal septicemia, air sacculitis, chronic respiratory diseases, cellulitis, pericarditis, peritonitis and salphingitis (Nolan et al., 2020). Outbreaks among flocks cause significant economic losses in the poultry industry due to carcass condemnation, reduced egg laying, morbidity, and mortality, as well as costs incurred for disinfection and antimicrobial treatment (Dziva and Stevens, 2008; Mellata, 2013; Graveline et al., 2014; Guabiraba and Schouler, 2015). Colibacillosis is thought to begin as a respiratory tract infection in broilers that inhale fecal dust contaminated with APEC (Dziva and Stevens, 2008; Guabiraba and Schouler, 2015). Due to a lack of consistent phenotypic and genotypic characteristics, the gold standard for APEC classification is the chicken lethality assay (Wooley et al., 2000; Collingwood et al., 2014; Mageiros et al., 2021). APEC strains, which encompass a large number of *E. coli* sequence types, are one of the subpathotypes of ExPEC (Extra intestinal pathogenic *E. coli*) together with uropathogenic, sepsis-associated and neonatal meningitis *E. coli* (Johnson et al., 2008). Several studies have suggested that APEC and human ExPEC strains share similar virulence-associated genes despite their sources of isolation, indicating the increased potential for zoonotic *E. coli* infections in humans (Johnson et al., 2008; Jakobsen et al., 2011). For example, a human pathogenic *E. coli* strain belonging to the O25b:H4-ST131 pandemic clonal group has been isolated from poultry, pigs, and cattle (Mora et al., 2010; Nicolas-Chanoine et al., 2014). Although they cause significant damage to broiler flocks, little is known about the ecological niche of the APEC strains. Do they live within the barn setting or do they colonize and later escape the intestinal niche? Many of the details surrounding colibacillosis infections remain a mystery.

The primary treatment option for colibacillosis is antimicrobial therapy (Sargeant et al., 2019). Many antimicrobials that are critical for human medicine are used in the global poultry industry to prevent and treat bacterial diseases (Landoni and Albarellos, 2015). Antimicrobials had been used for decades to enhance growth and prevent infection in food animals (Xiong et al., 2018), but this overuse of antimicrobials lead to the emergence of antimicrobial resistance (AMR) among bacterial pathogens and was thought to be a significant contributor to the AMR crisis in human clinical settings. Hence, the usage of antimicrobials as a growth promotor has now been banned in many countries (Maron et al., 2013; Government of Canada, 2018; Xiong et al., 2018; McMillan et al., 2019). *E. coli* are resident microbes in the intestinal flora of most food animals (Partridge et al., 2018). In the gut, they are exposed to billions of other bacteria and viruses which may harbor antimicrobial resistant mobile genetic elements. *E. coli* are highly promiscuous and may be a significant reservoir of AMR (Lugsomya et al., 2018; Chuppava et al., 2019). For example, *E. coli* that possess extended-spectrum β-lactamases (ESBL) are resistant to penicillins and certain cephalosporins (i.e., 3rd and 4th generations) and because many of the resistance genes are located on mobile genetic elements, ESBL *E. coli* are now globally disseminated (Thaden et al., 2016). Infections caused by ESBL *E. coli* are associated with higher economic costs, longer hospital stays, and increased mortality compared to non-ESBL *E. coli* (Tumbarello et al., 2007; Malande et al., 2019). Poultry are a known reservoir of ESBL *E. coli*, and transmission from livestock to humans has been proven *via* multi-locus sequence typing and whole genome sequencing (Leverstein-van Hall et al., 2011; Overdevest et al., 2011; Nagy et al., 2021). This all becomes more prominent since *E. coli* were recently identified as the #1 AMR-associated bacterial species causing morbidity and mortality in humans worldwide (Antimicrobial Resistance Collaborators, 2022).

Most APEC strains are also likely to form biofilms, an important factor contributing to increased persistence and survival of APEC within broiler farms (Reisner et al., 2006; Rodrigues et al., 2019). Biofilms are a physiological state where bacterial cells become irreversibly attached to surfaces and enclosed in a self-secreted extracellular polymeric matrix composed of proteins, polysaccharides, and nucleic acids (Hung et al., 2013; Sanchez-Vizuete et al., 2015). Growth in biofilms offers certain advantages to APEC including facilitating the exchange of AMR and virulence-associated genes *via* horizontal gene transfer between bacteria of same or different species (van den Bogaard and Stobberingh, 2000). In addition, the biofilm matrix provides protection for the embedded cells, resisting detrimental effects of antibiotics, disinfectants, and the host immune system, all of which makes it difficult to eradicate biofilm-related infections in clinical and food production settings (Reisner et al., 2006; Kester and Fortune, 2014; Rodrigues et al., 2019). Despite the increasing occurrence of AMR, bacteria seem to remain susceptible to disinfectants; the presence of multiple active ingredients with different mechanisms of action means that resistance is more unlikely to arise (Davin-Regli, 2012). However, disinfectants are often used incorrectly in practice; the use of inappropriate concentrations, expired products, or the inactivation of compounds due to the presence of inorganic matter on insufficiently cleaned surfaces are common errors. The exposure of bacteria to sub-inhibitory concentrations of disinfectants may result in reduced susceptibility (Soumet et al., 2016), which can cause significant problems for producers at multiple steps in the food production pipeline. Further, insufficient cleaning of barn surfaces or in meat packaging facilities may allow biofilm formation to occur on equipment. We feel it is important to understand the lifestyle of APEC strains to prevent dissemination of disease in birds and potentially to humans.

We isolated 94 *E. coli* from 12 different colibacillosis outbreaks on Saskatchewan farms and analyzed these isolates for biofilm formation, and resistance to 27 antimicrobial compounds and four disinfectants. We wanted to determine if biofilm formation and AMR could be correlated and if *E. coli* from diseased birds were different than *E. coli* from healthy birds.

## Materials and Methods

### Broiler Sampling

Four- to six-week-old broiler birds that were suspected of having colibacillosis were submitted to the Poultry Extension Team (PEX) at the Western College of Veterinary Medicine, University of Saskatchewan, as part of a routine analysis for the Saskatchewan broiler industry. In each case, the birds originated from local producers that had farms within a 2-h driving distance of Saskatoon. Members of the PEX performed necropsy on each bird and submitted organ samples for microbial isolation and diagnosis by MALDI-TOF mass spectrometry (Prairie Diagnostic Services^1^). If *E. coli* infection in the birds was confirmed, the PEX sent samples of the heart, liver, and spleen, previously stored at 4°C, to the White lab. At the same time, the PEX requested from the same producers that they submit 3–4 healthy broilers from the flocks where the diseased birds had originated. The cecal contents from these birds were subsequently sent to the White lab.

### *Escherichia coli* Isolation and Identification

Samples of the heart, liver, and spleen (diseased birds) or cecal contents (presumed healthy birds) were placed in 2 ml Eppendorf Safe-Lock tubes containing 1 ml of phosphate-buffered saline (PBS) and a 5-mm steel bead (Qiagen #69989) and were homogenized for 5 min at 30 Hz using a mixer mill (Retsch; MM400). Aliquots of the organ homogenates were spread on MacConkey Agar and colonies allowed to grow overnight at 37°C. Suspected *E. coli* colonies were confirmed *via* positive indole and negative Simmons-citrate biochemical tests. In total, we analyzed 56 disease-causing or “systemic” *E. coli* isolates and 38 healthy or “cecal” *E. coli* isolates, collected from 12 outbreaks over a 2-year period (Supplementary Table 1). *E. coli* isolates with visually distinct colony morphologies were selected for further analysis.

### Bacterial Growth and Media Conditions

Bacterial isolates were stored in 20% glycerol at –80°C for long-term preservation. Isolates were inoculated from frozen stocks onto Tryptic soy agar (TSA; Becton Dickinson) and were incubated for 18 h at 37°C. A single colony was inoculated into 5 mL of LB broth and grown overnight at 37°C with shaking at 200 rpm. Overnight cultures were used for all subsequent experiments.

### Curli and Cellulose Testing

Overnight cultures were standardized to an optical density of 1.0 at 600 nm. Four microliter aliquots were spotted on Congo Red Agar (20 μg/mL of Congo Red dye in 1% tryptone, 0.5% yeast extract, 1.5% agar; pH 7.2–7.4), to visualize the rdar morphotype and curli production (Collinson et al., 1991) and onto Calcofluor White agar [200 mg/L fluorescent brightener #28; (Sigma-Aldrich, St. Louis, MO, United States) in 1% tryptone, 0.5% yeast extract, 1.5% agar] to visualize cellulose production (Solano et al., 2002).

### Crystal Violet Assay

Crystal violet assays were performed using the method of Merritt et al. (2011), with details outlined previously (Liu et al., 2018). For each isolate, 20 μL of standardized inoculum was used to inoculate wells of different 96-well plates, containing 180 μL of three biofilm media: (1) 1/2 tryptic soy broth (TSB), (2) M63 media (ammonium sulfate, potassium monophosphate, and ferrous sulfate), and (3) brain heart infusion (BHI) broth. Each isolate was inoculated into six replicate wells and grown at 28°C for 24 h; negative control wells contained 180 μL of broth and were not inoculated. After 24 h, liquid was removed and each plate was washed twice with 200 μL of PBS. Bacterial cells and extracellular biofilm matrix were fixed by adding 200 μL of methanol to each well and incubating at room temperature for 15 min. The methanol was removed, and plates were left to air dry for 30 min, prior to staining with 200 μL of 0.2% crystal violet for 5 min. Excess crystal violet was rinsed from each well using distilled water. Plates were left to air dry before addition of 160 μL of 33% glacial acetic acid. The absorbance of the resulting solution was measured at 570 nm using a spectrophotometer. The classification system developed by Stepanović et al. (2000) was used to describe biofilm formation by each strain. The cut-off OD (OD_c_) was calculated as three standard deviations above the average OD of the negative control wells; this value was compared to the mean OD for each strain. The biofilm categories were strong (mean OD > 4*OD_*c*_); moderate (4*OD_*c*_ > mean OD > 2* OD_*c*_); weak (2* OD_*c*_ > mean OD > OD_*c*_); or none (OD_*c*_ > mean OD).

### Biofilm Formation in the MBEC Assay^®^

*Escherichia coli* biofilm formation and the measurement of disinfectant susceptibility was performed using the MBEC Assay^®^ biofilm inoculator 96-well plates (Innovotech Inc., Edmonton, AB, Canada) according to previously described method (Harrison et al., 2010). A standardized inoculum of 1.5 × 10^7^ CFU/mL was added to biofilm growth media to a final volume of 150 μL per well. The MBEC plate lid containing 96 polystyrene pegs was placed into the 96-well microtitre plate base, sealed with parafilm and incubated at 37°C for 24 h with slight rocking on a tilting platform shaker. After incubation, peg lids were removed from the base plate and washed twice with sterile PBS to remove loosely attached cells. Biofilm formation on pegs was quantified by viable cell counts following disruption of the biofilm by sonication for 30 min with a bath sonicator (Branson #3510, Canada). Dislodged biofilm cells were serially diluted and grown on Mueller Hinton (MH) agar plates (Liu et al., 2018; Sivaranjani et al., 2021).

### Determining the Minimum Inhibitory Concentration and Minimum Bactericidal Concentration for Disinfectants

The minimum inhibitory concentration (MIC) was determined by broth microdilution according to the Clinical and Laboratory Standards Institute (CLSI) standard for antimicrobial susceptibility testing (CLSI, 2020). Serial twofold dilutions of each disinfectant (Supplementary Table 2) were prepared in MH broth to a final volume of 90 μL per well. Ten μL aliquots of culture, representing 1.5 × 10^7^ bacterial cells, were added to each well, the plates were covered and incubated at 37°C with slight rocking. After 24 h of growth, the OD at 600 nm of each well was measured by an xMarkTM Microplate Absorbance Spectrophotometer (Bio-Rad, Mississauga, ON, Canada). The MIC was recorded as the concentration in the wells where bacterial growth was visibly inhibited. To determine the Minimum Bactericidal Concentration (MBC), 100 μl of aliquots from MIC wells were sub-cultured on MH agar and incubated for 24 h. The lowest concentration that resulted in no bacterial growth on MH agar plates was determined as the MBC.

### Disinfectant Susceptibility Testing

#### Planktonic Cells

Cells from overnight cultures were washed twice with sterile PBS and resuspended in 5 mL of fresh MH broth. The MH broth cultures were normalized to an optical density of 1.0 at 600 nm and the starting concentration of bacteria (CFU/mL) for each culture before disinfectant challenge was determined by viable cell counts. Serial twofold dilutions of each disinfectant were prepared in MH broth with a final volume of 90 μL in each well of a 96-well microtitre plate. Ten μL of inoculum was added to 90 μL of MH broth containing disinfectant and incubated at 37°C for 30 min. To test the viability of cells within each well, 10 μL aliquots were removed and sub-cultured into fresh MH broth and the remaining 90 μL was spread on a fresh MH agar plate. Both the sub-cultures were incubated at 37°C for 24 h. The lowest concentration resulting in no growth in liquid and on agar plates was recorded as the bactericidal concentration of disinfectant.

#### Biofilm Cells

After 24 h growth, biofilms formed on polystyrene pegs were transferred to a 96-well microtitre plate in which serial double dilutions of disinfectants were prepared in MH broth. Prior to disinfectant challenge, 6 control pegs were broken off from each plate, sonicated for 30 min in recovery media and the resuspended cells were serially diluted to determine the average starting number of biofilm cells for each strain. The remaining pegs were exposed to disinfectant for 30 min, rinsed twice in sterile PBS and cells were dislodged by bath sonication as described above. For each disinfectant concentration, cells from six replicate pegs were serially diluted and grown on MH agar plates to determine the number of viable cells remaining. The killing efficacy of disinfectants on biofilm cells was determined as the lowest concentration of disinfectant resulting in viable cell counts at or below the detection limit of 125 CFU per mL.

### Antimicrobial Susceptibility Testing

Antimicrobial susceptibility of all strains was determined *via* broth microdilution using the Gram-negative panel for the MicroScan system (Beckman Coulter, Mississauga, ON, Canada), testing the following 26 agents: amikacin, amoxicillin-clavulanic acid, ampicillin-sulbactam, ampicillin, aztreonam, cefazolin, cefepime, cefotaxime, cefoxitin, ceftazidime, ceftriaxone, cefuroxime, cephalothin, ciprofloxacin, doripenem, ertapenem, gentamicin, imipenem, levofloxacin, meropenem, nitrofurantoin, piperacillin-tazobactam, tetracycline, tigecycline, tobramycin and trimethoprim-sulfamethoxazole. Chloramphenicol, colistin, and nalidixic acid testing was performed using agar dilution (Wiegand et al., 2008). For each antibiotic, petri plates were prepared containing LB agar (lysogeny broth, 1.5% agar) plus antibiotic ranging from 0.5 μg/mL to 32 μg/mL. Isolates were streaked on blood agar (Oxoid #CM0055) and incubated for 18 h at 37°C. A 0.5 MacFarland standard was made for each isolate and isolates were spotted onto antibiotic plates using a 96-pin microplate replicator. Plates were incubated at 35°C for 24 h before reading MIC values. For quality control, *E. coli* ATCC 25922, *Staphylococcus aureus* ATCC 29213 and *Pseudomonas aeruginosa* ATCC 27853 were included as appropriate.

The results were interpreted according to the CLSI Guidelines (CLSI, 2020). MIC breakpoints were as follows: ampicillin (≥32 μg/mL), cefazolin (≥8 μg/mL), cephalothin (≥32 μg/mL), ceftazidime (≥16 μg/mL), ceftriaxone (≥4 μg/mL), cefuroxime (≥32 μg/mL), cefotaxime (≥4 μg/mL), cefepime (≥16 μg/mL), amoxicillin + clavulanic acid (≥32 μg/mL), ampicillin + sulbactam (≥32 μg/mL), cefoxitin (≥32 μg/mL), aztreonam (≥16 μg/mL), gentamicin (≥8 μg/mL), tobramycin (≥16 μg/mL), nalidixic acid (≥64 μg/mL), tetracycline (≥16 μg/mL), colistin (≥4 μg/mL), chloramphenicol (≥32 μg/mL), ciprofloxacin (≥1 μg/mL), doripenem (≥4 μg/mL), ertapenem (≥2 μg/mL), imipenem (≥4 μg/mL), meropenem (≥4 μg/mL), nitrofurantoin (≥128 μg/mL).

### Statistics

Graphing of data and statistical analyses were performed using GraphPad Prism software v.8.0.2 (San Diego, CA, United States). Data from the disinfectant susceptibility assays were logarithmically transformed and analyzed using the Kruskal–Wallis test with *post hoc* analysis *via* Dunn’s multiple tests. Statistical differences in biofilm biomass, as measured by crystal violet staining, were determined using an ordinary-one way ANOVA test [*F* = 14.28, *p* > 0.0001]. Distribution of systemic isolates was analyzed using a chi-square test to determine whether the frequency of positive biofilm formation in each media was statistically significant [chi-square statistic: 15.74, df = 2, *p* > 0.0001].

### Genomic DNA Extraction

Genomic DNA was extracted from each *E. coli* isolate using the GenElute Bacterial Genomic DNA Extraction Kit (Sigma-Aldrich, St. Louis, MO, United States), according to the manufacturer’s instructions. Purity of the DNA was assessed using a NanoDrop spectrophotometer (Thermo Fisher, Ottawa, ON, Canada) and quantified using a Qubit Fluorometer (Thermo Fisher Scientific, Ottawa, ON, Canada).

### Nanopore Sequencing

All *E. coli* isolates were sequenced on a Nanopore MinION according to the protocol developed by Lohman et al. (2018), with the amount of ligase added to each step calculated based on the expected number of DNA molecules available for sequencing. Small fragments were removed from the genomic DNA eluate by adding 0.4x magnetic beads to each sample (NucleoMag NGS beads, Macherey-Nagel, Allentown, PA, United States). DNA fragments larger than 500 bp were bound, washed in 80% ethanol and eluted in 1 mM Tris buffer, pH 8.0. DNA was stored at –20°C for up to 1 week before sequencing.

Up to 200 fmol of purified and size-selected DNA was added to a Lobind Eppendorf tube. Distilled water was added to a final volume of 30 μL. Nicks and gaps in the DNA were repaired using FFPE DNA Repair Mix (#M6630; New England Biolabs, Whitby, ON, Canada) and polyA-tails were added to DNA fragments using the Ultra II End Repair/dA-Tailing Module (#M7645; New England Biolabs). Nanopore barcodes (#NBD-104; Oxford Nanopore Technologies, Oxford, United Kingdom) were ligated to DNA fragments using Blunt/TA Master Mix (New England Biolabs). Multiplexed samples were pooled into a single Eppendorf tube and the reaction was adjusted to 1 M NaCl. DNA repair enzymes and excess DNA barcodes were removed using a 0.2X magnetic bead clean-up. Beads were washed twice with 80% EtOH and the DNA was eluted in 10 mM Tris, pH 8.0. Sequencing adapters (AMII; Oxford Nanopore Technologies EXP-NBD104) were ligated to the ends of DNA fragments using Quick T4 DNA ligase (New England Biolabs) before a final bead clean-up (1:1 ratio of beads to sample) was performed to remove T4 DNA ligase and excess sequencing adapters from the final purified DNA library. Beads were washed by incubating two times in long chain fragment buffer and resuspending by flicking. DNA was eluted in 1mM Tris Buffer, pH 8.0. The final library was prepared and loaded onto the sequencer as described in the SQK-LSK109 protocol. Sequencing was performed using R.9.4.1 MinION flow cells. Sequencing runs were terminated after approximately 24 h.

Base-calling and de-barcoding of Nanopore reads was performed using MinKNOW (version 2.0) and Guppy (version 2.1.1) software (Oxford Nanopore Technologies). Read quality and lengths for each sequencing run were determined and visualized using the NanoPack software (version 1.35.4) developed by De Coster et al. (2018). Additional read trimming was performed using Porechop to remove any barcodes missed by Guppy (Wick, 2017). Reads were filtered for quality using Nanofilt to ensure that only reads with a Q score of 10 or greater were included in draft assemblies (–q 10) (De Coster et al., 2018).

Rough draft genomes were assembled using Unicycler (version 0.4.8) (Wick et al., 2017). A summary of the characteristics of the 96 *E. coli* strains selected for draft genome assembly, as well as sequencing information can be found in Supplementary Table 1. Each isolate tested was considered distinct due to having a unique combination of biofilm and AMR profiles.

### Phylogroup Determination, Plasmid Identification, and Antimicrobial Resistance Gene Localization

The phylogroup of each *E. coli* strain was determined using the Clermontyping tool^2^, a web-based platform that predicts phylogroups based on an *in silico* quadruplex PCR to screen the whole genome FASTA sequence of an isolate for the *arpA*, *chuA, yjaA*, TspE4.C2 and TrpAgpC genes (Beghain et al., 2018).

We screened *E. coli* draft assemblies for plasmid sequences using PlasFlow, a program that uses a neural network to differentiate plasmid from chromosome sequences and has a higher accuracy than BLAST-based programs such as Plasmid Finder (Krawczyk et al., 2018). The draft chromosome and plasmid sequences were screened for antimicrobial resistance genes using the ResFinder database (ResFinder 4.0) from the Centre for Disease Epidemiology (Bortolaia et al., 2020). Genes were identified and used to generate presence and absence data for all 94 *E. coli* isolates. In the end, 28 known AMR genes were identified as being present in at least one isolate, encoding for resistance to tetracycline (*tetA, tetB*), sulfonamides (*sul1, sul2*), trimethoprim (*dfraS*), aminoglycosides [*aph(6)-Id, aph(3′′), aph(4)-I1a, aph (3′), aadA1, aac(2), aac(3)-IV, aac(3)-VI, aac(3)-2d*], and beta-lactam antibiotics (*bla*_TEM–18_, *bla*_TEM–176_, *bla*_TEM–105_, *bla*_CMY–2_, *bla*_CMY–138_, *bla*_CMY–14_, *bla*_CMY–149_, *bla*_CMY–15_, *bla*_CMY–16_, *bla*_CMY–155_, *bla*_CMY–122_, *bla*_CMY–4_, *bla*_OXA–1_). The *mdfA* gene encodes a multi-drug efflux pump.

## Results

### Disease-Causing *Escherichia coli* Are More Drug-Resistant Than Cecal *Escherichia coli*

A total of 94 *E. coli* strains were isolated from birds with colibacillosis (*n* = 56; systemic *E. coli*) and from presumed healthy birds of the same flock (*n* = 38; cecal *E. coli*). These *E. coli* strains represented 12 outbreaks on Saskatchewan farms between 2019 and 2020. Antimicrobial susceptibility testing was performed on all 94 isolates against 27 different antimicrobials: the results are summarized in Table 1. Resistance to Tetracycline (29.8%), Ampicillin (24.6%), Gentamicin (23.4%), and Tobramycin (19.1%) was the most observed. In contrast, there was no resistance detected to colistin, chloramphenicol, or the carbapenems. 43 isolates were susceptible to all 27 tested antimicrobials, while 32 were resistant to three or more drugs, and nine isolates were resistant to drugs from six different classes (Supplementary Table 1). Overall, the systemic isolates were significantly more drug resistant than the cecal isolates; 71% of systemic *E. coli* were resistant to at least one drug compared to only 29% of cecal *E. coli* (Supplementary Table 1). Tetracycline resistance was common in both groups, but resistance to ampicillin, gentamycin, and tobramycin was 20–30% higher in systemic isolates (Table 1). Systemic isolates also had higher MIC values for first and third generation cephalosporins, compared to cecal isolates (Table 1). Resistance to cefoxitin was found exclusively in systemic isolates, while Cefepime resistance was exclusively identified in cecal isolates.

**TABLE 1:** Minimum inhibitory concentration (MIC) distribution for systemic (*n* = 56) and cecal (*n* = 38) *E. coli* isolates cultured from Saskatchewan broilers.

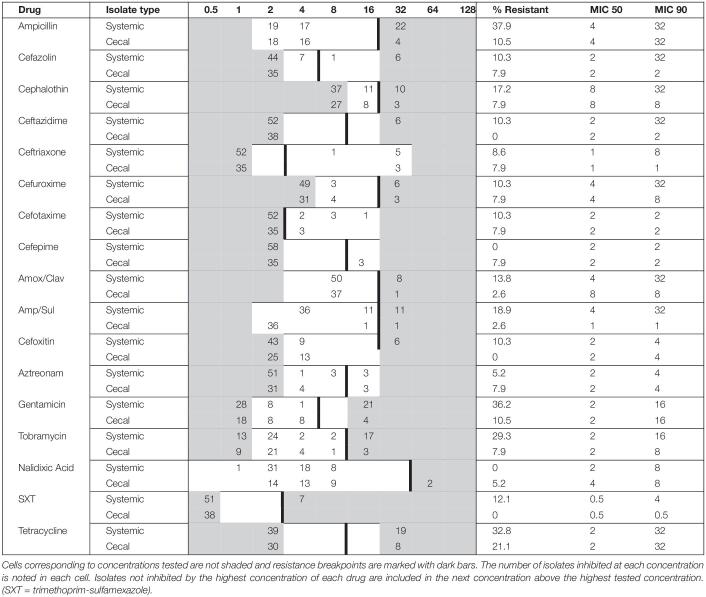

### Systemic Isolates Form Greater Biofilms in Rich Media

**FIGURE 1:**
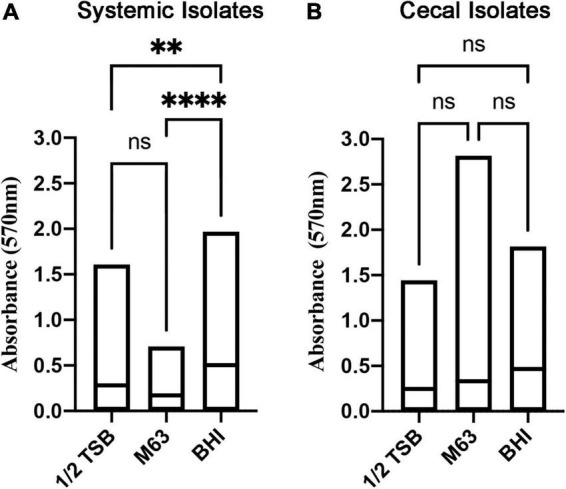
Quantitation of biofilm biomass for systemic and cecal *E. coli* isolates grown in three different growth media. Bars represent the mean absorbance of dissolved crystal violet that was used to stain biofilm cells and extracellular matrix from 56 systemic *E. coli* (A) or 38 cecal *E. coli* (B). Statistical significance between different growth media was tested by one-way ANOVA (^****^p < 0.0001; ^**^p < 0.01; ns, *p* > 0.05).

Biofilm formation is associated with bacterial persistence, and we wanted to determine if this physiological state was correlated with antibiotic resistance. All *E. coli* isolates were screened for biofilm-forming ability in three different liquid media: nutrient rich BHI broth versus relatively nutrient poor ½ TSB and M63 media. Crystal violet staining was used to quantify and classify the level of biofilm. Overall, the systemic isolates generated significantly more biomass in nutrient rich BHI broth, as compared to the other two media (Figure 1A), whereas the cecal isolates did not show a clear preference for any one media type (Figure 1B).

**FIGURE 2:**
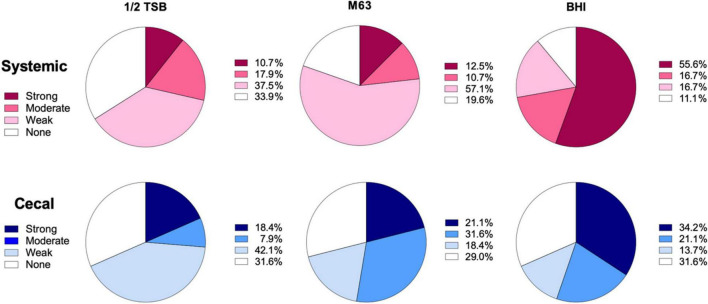
Classification of biofilm formation by systemic and cecal *E. coli* isolates grown in three different growth media. Biofilm formation in ½ TSB, M63, and BHI broth was classified using a formula where the cut-off OD (OD_*c*_) was set as three standard deviations above the average OD of the negative control wells: Strong = mean OD > 4*OD_*c*_; Moderate = 4*OD_*c*_ > mean OD > 2* OD_*c*_; Weak = 2* OD_*c*_ > mean OD > OD_*c*_; or None = OD_*c*_ > mean OD. Systemic isolates (*n* = 56) are represented by shades of red/pink while cecal isolates (*n* = 38) are represented by shades of blue.

To classify the level of biofilm formation for individual isolates, we used the formula described by Stepanović et al. (2000) (see section “Materials and Methods”). Isolates were categorized as positive if they formed strong or moderate biofilms, while isolates that produced none or weak biofilms were categorized as negative. 72% of systemic isolates were positive for biofilms in BHI broth, as compared to only 55% of cecal isolates (Figure 2). In contrast, 53% of cecal isolates were biofilm positive in M63 media, as compared to only 23% of systemic isolates (Figure 2). In general, systemic isolates were significantly worse at forming biofilms in nutrient poor media, with 28% positive in ½ TSB and 23% positive in M63 media (*X*^2^, *p* < 0.005) (Figure 2). In contrast, a roughly equal proportion of cecal isolates formed biofilms in M63 (53%) as BHI broth (55%), suggesting that these strains are equally capable of forming biofilms in nutrient-poor and nutrient-rich conditions (Figure 2). Approximately 30% of cecal isolates formed no biofilm in each media tested (Figure 2).

We also screened all *E. coli* isolates for curli and cellulose production since these polymers are central to biofilm formation in the host and the environment (White et al., 2011; MacKenzie et al., 2017; Chuppava et al., 2019). Sixty-two isolates produced both curli and cellulose, distributed as 73% (41/56) of systemic isolates and 55% (21/38) of cecal isolates (Supplementary Table 1). Twenty isolates produced only curli, consisting of 10 systemic and 10 cecal isolates. Nine isolates produced only cellulose, consisting of five systemic and four cecal isolates, and five cecal isolates were the only strains negative for both curli and cellulose. Taken together, the results indicated that systemic isolates had a slight increase in biofilm phenotypes as compared to cecal isolates.

### Identifying the Strongest Biofilm Forming *Escherichia coli* Isolates and Testing Their Resistance to Commercial Disinfectants

**FIGURE 3:**
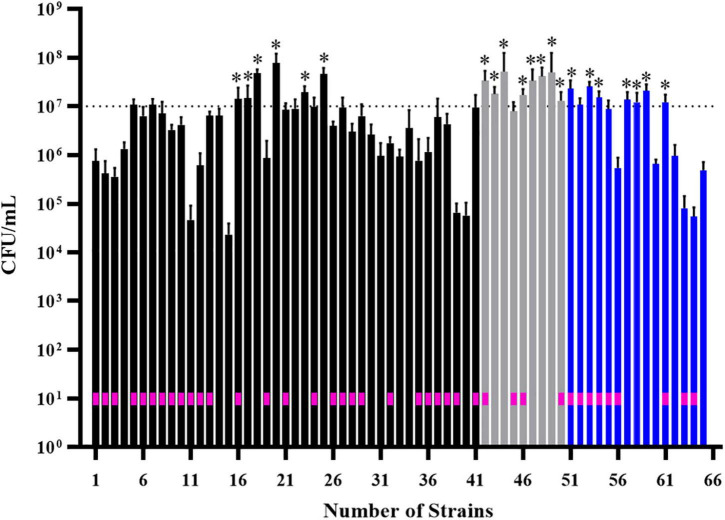
Screening of 55 biofilm-positive *E. coli* isolates for their ability to form biofilms on polystyrene pegs. After growth for 24 h at 37°C in BHI (black bars), M63 (gray bars) or ½ TSB (blue bars) media, cells were dislodged and enumerated from each peg (*n* = 6); histogram bars represent the average CFU/mL values and error bars represent the standard deviations. Some isolates formed strong biofilms in more than one media, which is why 65 bars are shown on the graph. Stars above the bars denote the isolates with cell densities greater than 10^7^ CFU/mL; these were considered the strongest biofilm forming isolates (*n* = 21). The *E. coli* strains isolated from diseased birds are designated by a pink box within the corresponding histogram bar.

We performed additional screening of 55 isolates that were classified as strong biofilm formers in BHI, M63 and ½ TSB media (Supplementary Table 1). We tested the ability of strains to adhere and form biofilms on polystyrene pegs in MBEC biofilm inoculator plates. Rather than measure biomass by staining, we focused on live cell counts, looking for strains with cell counts greater than 10^7^ CFU, which we considered an appropriate cut off for good biofilm formation, based on previous MBEC assays (Liu et al., 2018). In total, 21 isolates formed biofilms with cell counts above 10^7^ CFU (Figure 3; stars). consisting of 11 systemic and 10 cecal *E. coli*.

We further screened 12 of these isolates (7 systemic + 5 cecal) for resistance to four commercial disinfectants (Supplementary Table 2). Each disinfectant was able to kill planktonic cells of all *E. coli* isolates at low concentrations, ranging from 0.0016-0.0031% for Virocid, 0.125-0.25% for Virkon, 1.9–3.8 ppm for the quaternary ammonium disinfectant DDAC and 0.031–0.063% for H_2_O_2_ (Supplementary Table 3). The MBC values of all four disinfectants were either equal or one serial dilution higher than the corresponding MIC values. We deemed these results to be somewhat misleading because MIC and MBC determination requires exposure to test antimicrobials for 24 h, whereas most common disinfectants require contact times of only minutes to achieve bactericidal effects. Therefore, we repeated disinfectant screening on planktonic and biofilm cells of each of the 12 *E. coli* strains with a 30 min exposure. The starting cell numbers for each strain ranged between 10^6^ and 10^8^ CFU (Supplementary Table 4). Virkon and Virocid disinfectants exhibited effective bactericidal activity against both cell types of all 12 isolates even at low concentrations (Table 2 and Supplementary Figures 1, 2). The concentration of Virocid required to achieve full bactericidal effect was 1/2- to 1/32- fold lower than the suppliers’ recommended concentration, and for Virkon it was reduced approximately by 1/2- to 1/8- fold (Table 2 and Supplementary Table 2). DDAC was effective at killing planktonic cells of the 12 *E. coli* isolates with bactericidal concentrations ranging from 4.7 to 9.4 ppm (Table 2). However, biofilm cells of all 12 isolates had 2- to 64-fold increased resistance to DDAC, and one of the systemic isolates was not killed at the manufacturer recommended concentration of 300 ppm (Table 2 and Supplementary Figure 3). Higher concentrations of DDAC were required to eradicate the biofilms of three systemic isolates as compared to the five cecal isolates (Table 2). The results with H_2_O_2_ were similar to DDAC, as planktonic cells of all tested isolates were susceptible to low concentrations, but biofilms cells had 4- to 64-fold increased resistance (Table 2 and Supplementary Figure 4). Again, 4 systemic isolates were more resistant and required 4% H_2_O_2_ for the complete eradication of biofilms, whereas all five cecal isolates were susceptible to concentrations of 2% or less (Table 2 and Supplementary Figure 4). To determine if these trends based on isolate source were consistent, we would need to analyze more *E. coli* strains.

**TABLE 2:** Bactericidal effect of disinfectants on planktonic and biofilm cells within 30 min of contact time.

Strain #	Strain ID	Bactericidal effect of disinfectants concentrations on planktonic cells (PC) and biofilm cells (BC)							
		Virocid %		Virkon (%)		DDAC (ppm)		H_2_O_2_ (%)	
		PC	BC	PC	BC	PC	BC	PC	BC
**Systemic *E. coli* isolates**									
1	9226-3H1	0.016	0.125	0.25	0.5	9.4	300	0.063	2
2	4957-2L3	0.008	0.008	0.25	0.5	9.4	37.5	0.063	0.5
3	6245-2H3	0.016	0.125	0.25	0.5	9.4	300^#^	0.063	4
4	9619-1H2	0.008	0.008	0.25	0.5	9.4	18.8	0.063	4
5	6041-3L1	0.016	0.031	0.25	0.5	9.4	75	0.063	4
6	9619-3L1	0.016	0.125	0.25	0.5	9.4	75	0.063	4
7	9413-1S2	0.008	0.016	0.25	0.25	9.4	150	0.063	2
**Cecal *E. coli* isolates**									
8	6245-C2	0.016	0.063	0.25	0.5	9.4	75	0.063	1
9	4957-C5	0.016	0.016	0.25	0.5	4.7	18.8	0.063	0.25
10	6245-C5	0.016	0.031	0.25	0.5	9.4	75	0.063	1
11	0012-C1	0.008	0.016	0.25	0.5	9.4	75	0.063	0.5
12	6041-C2	0.016	0.016	0.25	0.25	9.4	75	0.063	2

*^#^The minimum recommended concentration was not enough to achieve the bactericidal effect on the biofilm cells.*

### Trends in Antimicrobial Resistance by Outbreak

As a final comparison of the *E. coli* strains analyzed, we divided each group of isolates from outbreak to outbreak. This perhaps has more biological relevance than comparing the large groups of systemic and cecal strains to each other, since in almost all cases outbreaks occurred on different poultry farms. We identified 20 unique antibiotic resistance profiles across 12 Saskatchewan colibacillosis outbreaks (Supplementary Table 1). The most common resistance pattern was ampicillin + gentamicin + tobramycin, which was identified in isolates from 7 of 12 outbreaks. In all but three outbreaks, we identified systemic *E. coli* isolates that were resistant to at least four drugs. While the cecal isolates were generally susceptible, we identified at least one drug resistant cecal isolate in half of the outbreaks. In two outbreaks, cecal isolates were identified that were resistant to more than four drugs. This comparison again revealed an overall trend for systemic *E. coli* isolates to be more antibiotic resistant than cecal isolates, but there was a lot of diversity and no absolute patterns.

### Draft Genome Sequencing and Phylogeny of Systemic and Cecal *Escherichia coli* Isolates

For each outbreak, disease-causing *E. coli* were isolated from up to three diseased birds and cecal *E. coli* were isolated from up to three healthy birds from the same flocks. Strain profiles based on the combination of curli, cellulose and AMR phenotype screening, indicated that the 94 isolates did not consist of groups of identical clones (Supplementary Table 1).

We determined the phylogroup of each *E. coli* strain using the Clermontyping tool (see footnote 2). Systemic isolates were primarily distributed into phylogroups G (32.8%), A (20.7%), and D (18.9%) (Supplementary Table 1). This was consistent with extraintestinal pathogenic *E. coli*, with phylogroups G and D known to contain many pathogenic *E. coli* strains (Clermont et al., 2000, 2019). In contrast, the majority of Saskatchewan cecal strains were distributed in phylogroups A (60.5%) and B1 (15.8%) (Supplementary Table 1), which are phylogroups known to typically contain commensal and environmental *E. coli* isolates (Duriez et al., 2001). The wide genetic diversity identified in the pool of systemic isolates suggests that transmissible elements, such as plasmids, may play a role in infection (Sola-Gines et al., 2015).

### Known Antimicrobial Resistance Genes Appear to Be Concentrated on Plasmids of Systemic and Cecal *Escherichia coli*

The *E. coli* Nanopore draft assemblies were analyzed for plasmid sequences using PlasFlow (Krawczyk et al., 2018). We found that the 56 systemic isolates possessed an average of four plasmids (range from 0 to 9), while 38 cecal isolates possessed an average of 1 plasmid (range from 0 to 4) (Supplementary Figure 5). Next, we screened draft chromosome and plasmid sequences for antimicrobial resistance genes to see if we could identify genes unique to either group of isolates. We used the publicly available ResFinder 4.0 database from the Centre for Disease Epidemiology (Bortolaia et al., 2020). Due of the high error rates of Nanopore sequencing, and a lack of any polishing or consensus step in the Unicycler assembly program, we did not search for AMR acquired *via* point mutations.

**FIGURE 4:**
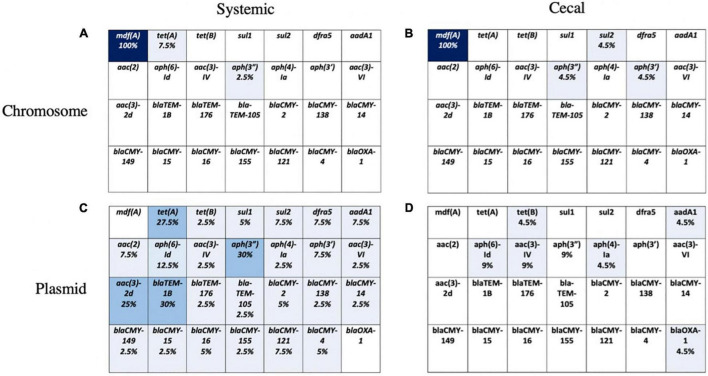
Presence of 28 known AMR genes on chromosome and plasmid for systemic and cecal *E. coli* strains. Percent abundance of AMR genes on the chromosome of systemic *E. coli* **(A)** and cecal *E. coli* **(B)** or the plasmids of systemic *E. coli* **(C)** and cecal *E. coli* **(D)**. Light blue represents a proportion greater than 10%, medium blue = 10–30%, and dark blue represents greater than 30%.

The chromosomal and plasmid DNA sequences for each isolate were screened for the presence of 28 known AMR genes. We identified few chromosomal antimicrobial resistance genes in our *E. coli* population, except for *mdfA*, an outer membrane transporter that confers resistance to quaternary ammonium compounds, the active ingredient in a common commercial barn disinfectant. A small number of systemic isolates had chromosomal *TetA* genes, and four cecal isolates had chromosomal aminoglycoside or sulfonamide genes (Figures 4A,B). The remaining AMR genes identified in both groups were present on plasmids (Figures 4C,D). Over 25% of systemic isolates possessed plasmid-encoded tetracycline [*tet(A)*], beta-lactam (*blaTEM-1B*), and aminoglycoside [*aac(3)-*2d, *aph(3”)*] resistance genes (Figure 4C). Plasmid-mediated aminoglycoside resistance [*aph(6)-Id, aac(3)-IV]* was the most common type found in cecal isolates, at 9% abundance (Figure 4D). Almost half (41%) of cecal isolates lacked any plasmid-encoded AMR genes, compared to only 10% of systemic isolates.

## Discussion

In this study, we analyzed *E. coli* isolates from colibacillosis outbreaks on Saskatchewan broiler farms. The comparison between 56 systemic or disease-causing *E. coli* strains and 38 cecal isolates from presumed healthy birds in the same flocks revealed an overall trend where the systemic isolates had a greater degree of AMR and were more likely to form biofilms than cecal isolates. This trend was not absolute and there were exceptions depending on the outbreak. The patterns of AMR and biofilm formation tended to be unique within each outbreak and between systemic and cecal isolates within each outbreak. Overall, there was a lot of phenotypic diversity, as reported by others (Schouler et al., 2012; Clermont et al., 2013; Riley, 2014; Cordoni et al., 2016). The differences observed between the systemic and cecal *E. coli* isolates indicated that they likely represent distinct groups of *E. coli* within the broiler farm environment. If the cecal *E. coli* were the source of disease, we would have expected to identify conserved AMR patterns within each outbreak, but we did not. Draft genome sequencing indicated that there were differences in plasmid content between the two groups of strains analyzed. Understanding the biological reasons that systemic and cecal *E. coli* are different is difficult without knowing the exact pressures that these groups of *E. coli* are subject to (Reisner et al., 2006; Brzuszkiewicz et al., 2009; Coombes, 2009; Azevedo et al., 2016; O’Boyle and Roe, 2021).

One of the goals for performing phenotypic analysis was to determine if there was a correlation between the degree of AMR and the biofilm-forming ability of individual *E. coli* strains, regardless of source. Despite the trend for systemic isolates having higher levels of antibiotic resistance and slightly more biofilm formation, there was no statistically significant relationship between AMR status and biofilm production in either the systemic or cecal group of *E. coli* strains analyzed. A roughly equal proportion of systemic and cecal isolates formed strong biofilms despite systemic isolates being more drug resistant. Nine of 11 systemic isolates that formed the strongest biofilms in 96-well plates and on polystyrene pegs were multi-drug resistant; the remaining two systemic isolates and 10 cecal isolates that formed strong biofilms were susceptible to all tested antimicrobials. This indicated that there was no direct correlation between the two phenotypes. Curli and cellulose production were both prevalent, even though there were a group of isolates (cecal) (roughly 30%) that did not form biofilms in any media.

Despite biofilm formation not being correlated with increased AMR, it is well established that this growth state is important for other aspects of persistence and survival (Harms et al., 2016; Jung et al., 2019; Yan and Bassler, 2019). The biofilm environment offers bacteria a source of nutrients as well as protection from external stressors such as desiccation, antimicrobials, and disinfectants. In our study, systemic isolates formed significantly more biofilms in BHI broth than in any other media, with 72% of isolates forming a positive biofilm. In contrast, less than 25% of systemic strains formed a positive biofilm in relatively nutrient-poor media. The significance of the media type is not fully understood, but is a little surprising since previous studies have shown that APEC strains form negligible biofilms in most media (Skyberg et al., 2007; Nielsen et al., 2018). However, the strains characterized in those studies were obtained from geographically diverse collections of APEC strains isolated from birds with various manifestations of disease. To our knowledge, our study is the first to analyze presumed APEC strains isolated exclusively from birds with confirmed sepsis in a small geographic area. Perhaps the *E. coli* strains responsible for a majority of systemic colibacillosis cases in Saskatchewan outbreaks possess genetic characteristics that favor biofilm formation in nutrient-rich media (Mellata et al., 2010, 2012). In contrast, for cecal isolates, there was no significant preference for the type of biofilm media, with approximately 50% of cecal isolates producing a biofilm in two media: M63 and BHI broth. This is consistent with previous reports that *E. coli* isolates of fecal origin can form biofilms in both minimal and nutrient-rich media (Skyberg et al., 2007; Nielsen et al., 2018).

Cleaning with disinfectants is one the most practical intervention strategies to eliminate bacterial contamination in the poultry barn setting. Many disinfectants are used in the poultry industry, with active ingredients belonging to aldehydes (e.g., formaldehyde), halogens (e.g., sodium hypochlorite), surface active agents [e.g., quaternary ammonium compounds (QAC)], oxidizing agents (e.g., H_2_O_2_), phenols and alcohols (Sagripanti and Bonifacino, 2000). The continuous exposure of residual disinfectants at lower concentrations may increase bacterial tolerance (Wieland et al., 2017). Any increase in tolerance could increase the bacterial adaptive resistance to antibiotics and enhance their survival fitness against various environmental stresses (Morente et al., 2013). We used the Calgary biofilm device to evaluate the killing efficacy of four commonly used disinfectants on the adherent population of cells from 12 of the strongest biofilm forming isolates. Each of the selected disinfectants is formulated with single or multiple active ingredients. Virkon is a solution formulated with multiple active ingredients including peroxygens and surfactants. It kills bacteria by oxidizing sulfur bonds in proteins and enzymes, resulting in cell wall rupture. Virocid is also a broad-spectrum disinfectant, formulated with synergistic blend of two different QAC (i.e., single chain and twin chain quaternary ammonia) along with glutaraldehyde and isopropanol. QACs interact with negatively charged cell membranes to elicit bactericidal action, while glutaraldehyde synergistically interacts with functional thiol and amine groups of proteins (Gilbert and Moore, 2005; Masadeh et al., 2013). It is believed that the interaction of active components with matrix proteins may facilitate the effective penetration of biofilm and synergistically kills the embedded bacterial cells (Osland et al., 2020). Our results showed that Virocid and Virkon had excellent killing at low concentrations against the planktonic and biofilm cells of all test strains, presumably due to the synergistic blend of multiple ingredients with non-specific bactericidal modes of action. In contrast, DDAC and H_2_O_2_ showed a large gap in killing where up to 64-fold higher concentrations were necessary to eradicate biofilm cells. The germicidal action of DDAC depends on numerous factors including the length of the *N*-alkyl chain, their combination with other active ingredients, pH, concentration used and the different target pathogens (Gilbert and Moore, 2005; He et al., 2013). H_2_O_2_ is a strong oxidizing agent that has been shown to damage bacterial DNA, proteins, and cellular membranes (Bell et al., 1997). It is well-known that bacteria residing in the inner matrix of a biofilm are protected from bactericidal concentrations of chemical agents, providing prime opportunities to develop resistance. Several studies have implicated that the sub-inhibitory concentrations of QACs may have a potential to select the emergence of AMR among pathogens, which could be raised from cross-resistance between QACs and other medically important antibiotics such as ampicillin, ceftazidime, cefotaxime, and ciprofloxacin (Nhung et al., 2015; Soumet et al., 2016; Nasr et al., 2018). Wieland and coworkers reported that the regular use of DDAC at low residual concentration would be able to select antibiotic resistance among *E. coli* and *Enterococcus* spp., which was confirmed by correlating the increased MIC values of DDAC with increased MIC values for several antibiotics, and high-level resistance was observed against aminoglycoside in enterococci (Wieland et al., 2017). These reports are in line with our results, that most of our isolates showed relatively common resistance to aminoglycoside and several other antibiotics. There was also a clear difference in susceptibility between systemic and cecal biofilms, as systemic isolates required higher concentration of DDAC and H_2_O_2_ for complete killing of biofilm cells. Our results suggest that the disinfectants with multiple active ingredients with different modes of action (i.e., Virocid and Virkon) are more effective against both systemic and cecal biofilms than the disinfectants with single mode of action (DDAC and H_2_O_2_).

Resistance to therapeutic antibiotics among *E. coli* isolated from poultry is of serious concern for both human and veterinary medicine, especially since it was just recognized that *E. coli* is the #1 cause of bacterial deaths due to resistant infections (Antimicrobial Resistance Collaborators, 2022). Recently, Canada has restricted the use of medically important antibiotics in food animals from being used as a growth promoter and requiring producers to obtain a veterinary prescription to treat infections in their flocks (Government of Canada, 2018). However, studies suggest that resistant bacteria can persist in the environment and spread AMR genes to commensal bacteria or between premises even with declining or no antimicrobial usage (Ge et al., 2005; Agunos et al., 2013). Reasons for this fitness advantage are likely to be complex but not limited to horizontal transmission of AMR genes between bacteria, improper cleaning and disinfection, biofilm formation and co-selection of resistance to certain antimicrobials. 71% of systemic isolates were resistant to between one and nine tested antimicrobial compounds. In contrast, only 29% of *E. coli* recovered from the cecal contents of uninfected birds were drug resistant or multi-drug resistant (MDR). Previous studies have demonstrated that resistance to specific drugs is correlated with the presence of ExPEC virulence genes (Johnson et al., 2006, 2012). Resistance to trimethoprim-sulfamexazole was exclusive to systemic isolates – this drug is significantly associated with the possession of the *afa* gene (Johnson et al., 2012). Afa is a fimbrial adhesin which is found in uropathogenic *E. coli* (UPEC) and is associated with recurrent infection (Bien et al., 2012). Most *E. coli* strains tested in our study demonstrated resistance to Ampicillin, Tetracycline, Gentamycin and Tobramycin, and displayed MDR as classified by resistance to as many as three different antimicrobial classes. These resistance trends are comparable to those of previous reports, with most clinical *E. coli* isolates recovered from either diseased or uninfected chickens exhibiting resistance to aminoglycosides, tetracyclines and sulfa drugs (Sáenz et al., 2003; Yang et al., 2004; Zhao et al., 2005; Varga et al., 2019).

Despite the presence of *E. coli* from diverse phylogroups, similar AMR profiles appeared in multiple outbreaks, suggesting that mobile genetic elements could be playing a role in resistance. To this end, draft genome sequencing revealed that numerous antimicrobial resistance genes were carried on plasmids. *E. coli* isolates with the same 9 drug resistance profiles were isolated from four outbreaks, while resistance to ampicillin, gentamicin, and tobramycin was present in over 50% of outbreaks. This may be indicative of transmission between farms, though further genetic characterization would be required to confirm this. Given the small geographic location of our study population and only two hatchling suppliers servicing central Saskatchewan, vertical transmission of APEC may also play a role in some of the similarities observed between outbreaks (Giovanardi et al., 2005). These systemic isolates may establish biofilms in the broiler barn environment and act as reservoirs for virulence and AMR genes in future outbreaks. *E. coli* can survive for years in the barn environment, particularly in dust (Schulz et al., 2016), perhaps supporting the prevailing hypothesis that colibacillosis infections occur *via* the inhalation of contaminated dust (Kabir, 2010). We also found that *E. coli* from Saskatchewan colibacillosis outbreaks had outbreak-specific media preferences for biofilm formation, suggesting that outbreak isolates may share properties which influence biofilm formation in a particular media type (Skyberg et al., 2007; Nielsen et al., 2018). In some cases, systemic and cecal isolates from a single outbreak exclusively formed biofilms in only a single media type. However, analysis of more outbreaks and a greater number of isolates is required to see if any trends are consistent. While biofilm preference was not correlated with any particular AMR profile, previous studies have identified biofilm formation as a key tool for the persistence of AMR genes in broiler barns (Zhai et al., 2020). The genetic components that promote biofilm formation in different media types may be linked to survival in the presence of different disinfecting agents (Zhai et al., 2020). A survey of the cleaning and disinfecting strategies used by Saskatchewan producers would help us to understand the role that biofilm formation plays in the persistence of outbreak-specific *E. coli* populations.

Proper biosecurity is critical for the management of colibacillosis on broiler farms. While biofilm formation and AMR profiles are not directly correlated, both likely play a role in the survival and pathogenicity of systemic isolates on farms. Outbreak tracking may provide a better information about the population of *E. coli* causing colibacillosis in a particular area. Bacterial persistence in the environment plays a key role in other recurrent bacterial infections, and tracking outbreaks may make it possible to identify *E. coli* reservoirs (Da Silva and De Martinis, 2013; Liao et al., 2021).

## Data Availability Statement

The raw data supporting the conclusions of this article will be made available by the authors, without undue reservation.

## Author Contributions

MS, MCM, JRD, JER, and APW: conceptualization, formal analysis, and writing—review and editing. MS, MCM, MKS, and LW: investigation. JER and APW: resources. MS, MCM, and APW: data curation and writing—original draft preparation. All authors: read and agreed to the published version of the manuscript.

## Conflict of Interest

The authors declare that the research was conducted in the absence of any commercial or financial relationships that could be construed as a potential conflict of interest.

## Publisher’s Note

All claims expressed in this article are solely those of the authors and do not necessarily represent those of their affiliated organizations, or those of the publisher, the editors and the reviewers. Any product that may be evaluated in this article, or claim that may be made by its manufacturer, is not guaranteed or endorsed by the publisher.

## Footnotes

1
http://pdsinc.ca/

2
http://clermontyping.iame-research.center/
