# Supplementary material for: Biofilm Formation and Antimicrobial Susceptibility of E. coli Associated With Colibacillosis Outbreaks in Broiler Chickens From Saskatchewan
Source: Front Microbiol. 2022 Jun 17;13:841516. doi: 10.3389/fmicb.2022.841516 (PMC9247541; doi:10.3389/fmicb.2022.841516)
Supplement: Supplementary file 1 [file Data_Sheet_1.pdf]

## Supplementary figures and tables

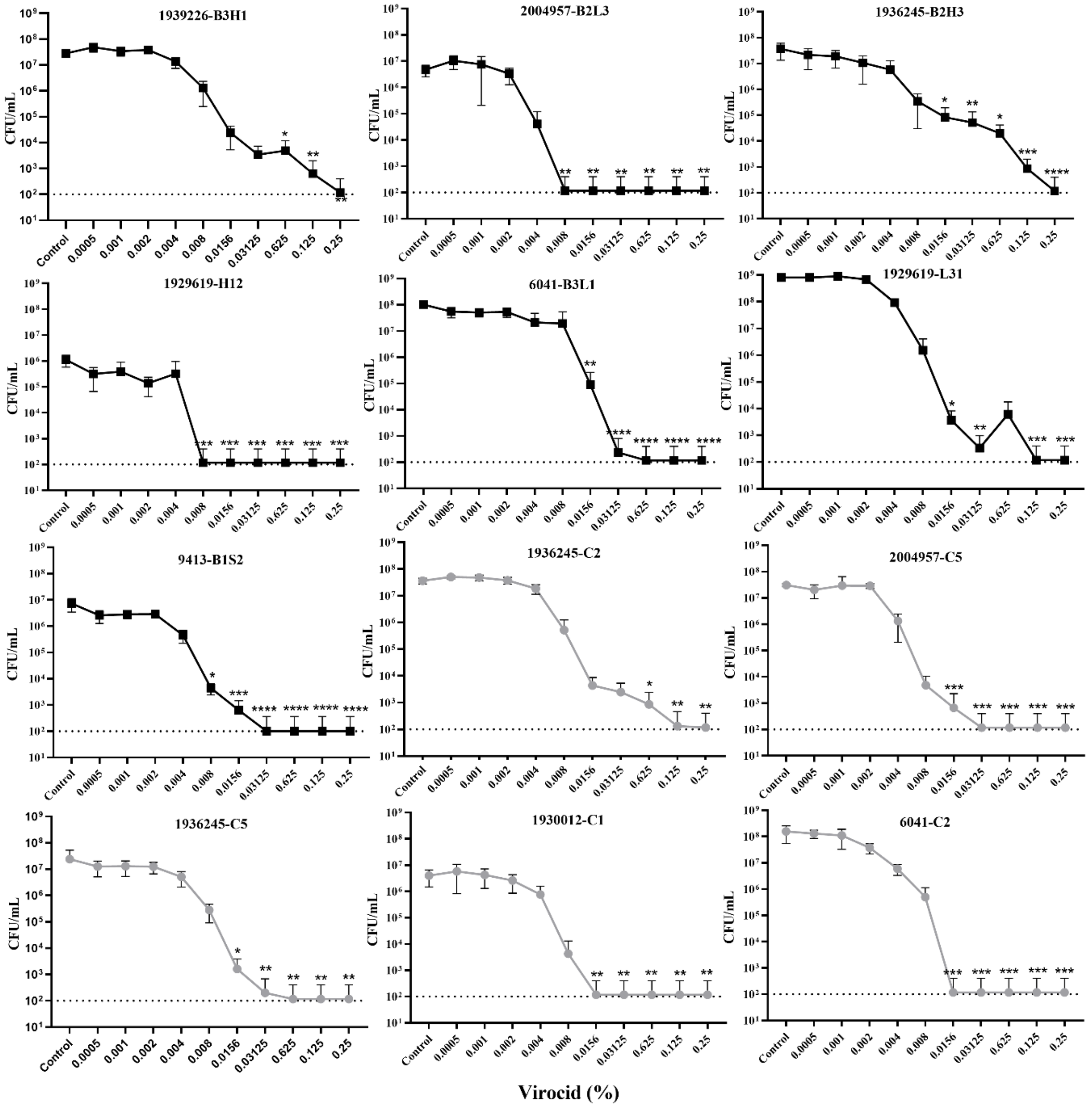


**Figure S1** Determination of viable cells after Virocid treatment on systemic (Black line) and cecal (Grey line) *E. coli* biofilms. Viable cells from each peg (n = 6) were enumerated after biofilm growth for 24 h and following Virocid exposure for 30 min. Symbols on the graph represent the mean ± SD from three independent experiments. Statistical significance is represented as follows: *P < 0.05; **P < 0.005; ***P < 0.0005; ****P < 0.0001.

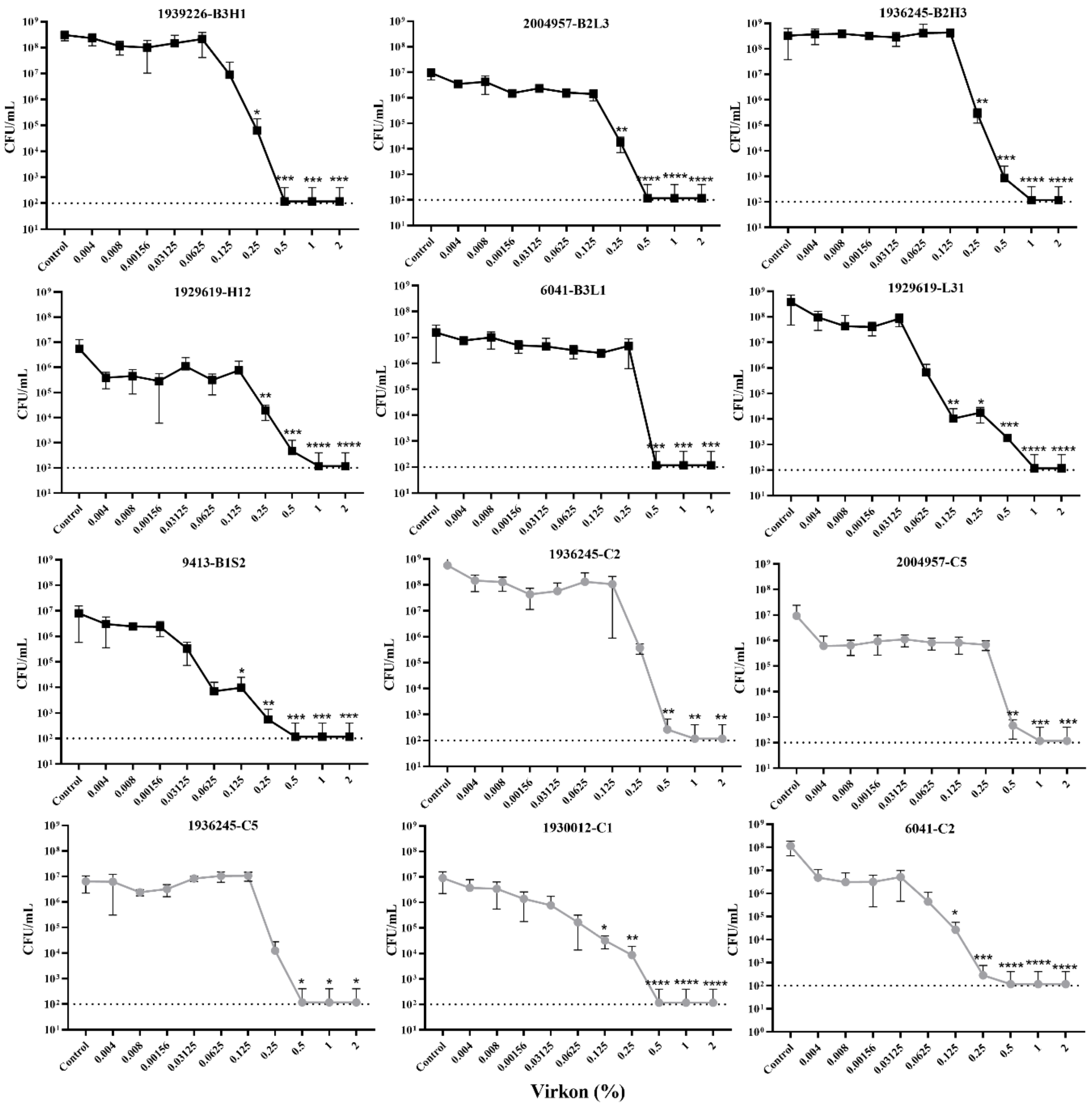


**Figure S2** Determination of viable cells after Virkon treatment on systemic (Black line) and cecal (Grey line) *E. coli* biofilms. Viable cells from each peg (n = 6) were enumerated after biofilm growth for 24 h and following Virkon exposure for 30 min. Symbols on the graph represent the mean ± SD from three independent experiments. Statistical significance is represented as follows: *P < 0.05; **P < 0.005; ***P < 0.0005; ****P < 0.0001.

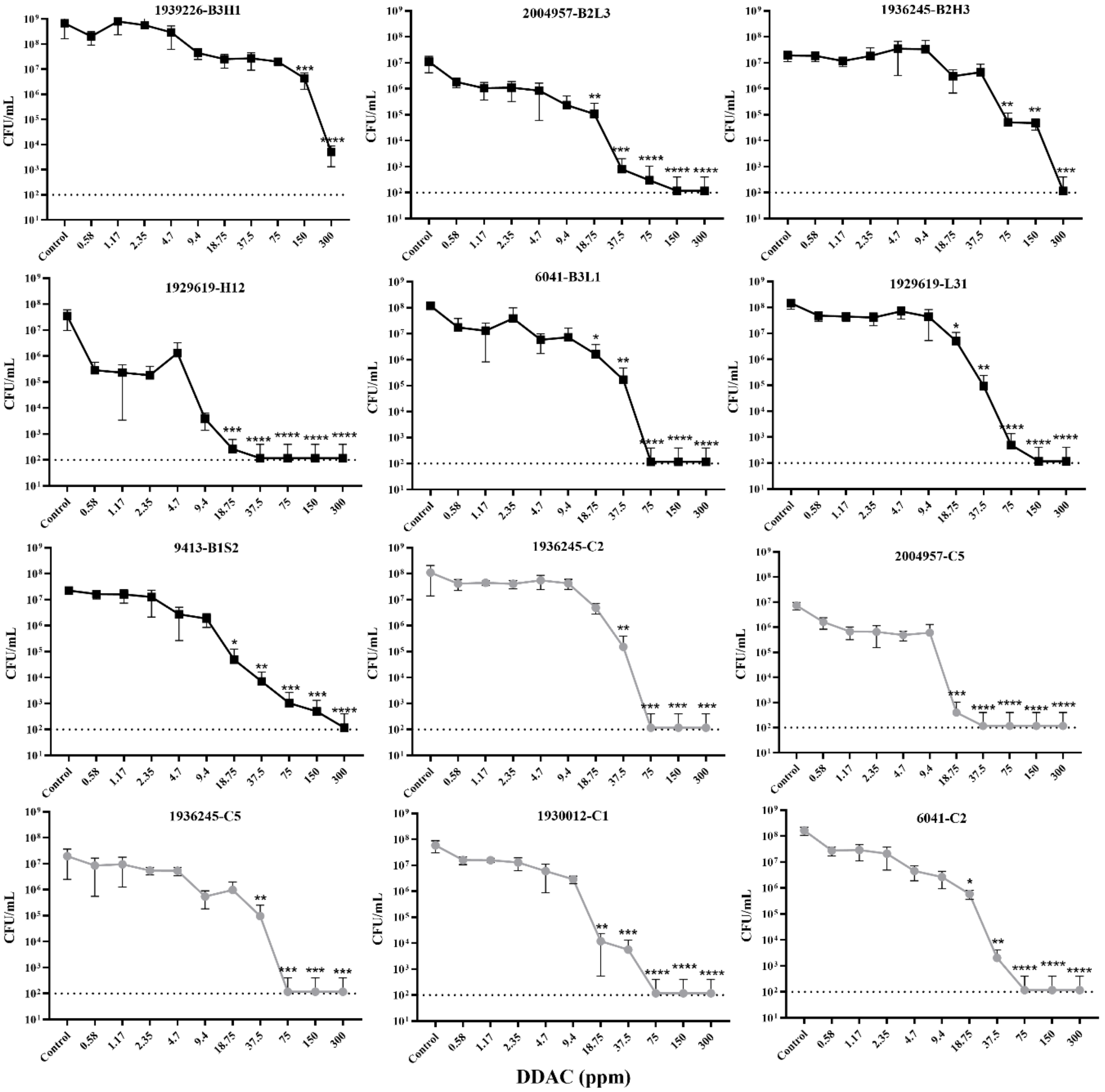


**Figure S3** Determination of viable cells after DDAC treatment on systemic (Black line) and cecal (Grey line) *E. coli* biofilms. Viable cells from each peg (n = 6) were enumerated after biofilm growth for 24 h and following DDAC exposure for 30 min. Symbols on the graph represent the mean ± SD from three independent experiments. Statistical significance is represented as follows: *$P < 0.05$; **$P < 0.005$; ***$P < 0.0005$; ****$P < 0.0001$.

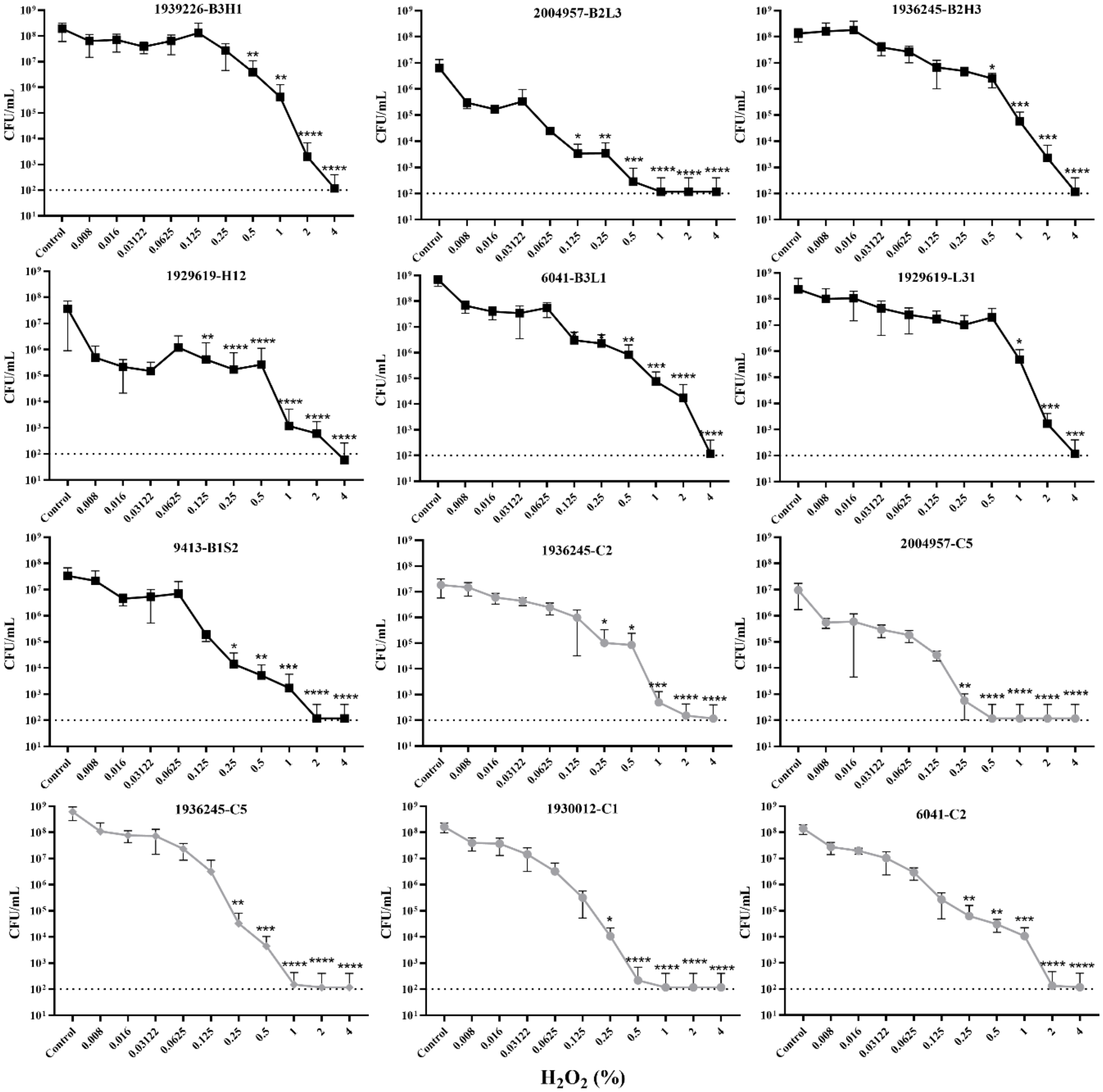


**Figure S4** Determination of viable cells after $H_2O_2$ treatment on systemic (Black line) and cecal (Grey line) *E. coli* biofilms. Viable cells from each peg (n = 6) were enumerated after biofilm growth for 24 h and following $H_2O_2$ exposure for 30 min. Symbols on the graph represent the mean ± SD from three independent experiments. Statistical significance is represented as follows: *P < 0.05; **P < 0.005; ***P < 0.0005; ****P < 0.0001.

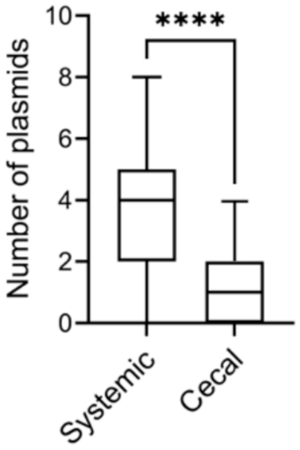


**Figure S5. Plot of average plasmid number per for 94 *E. coli* isolates from Saskatchewan poultry.** Plasmid prediction was performed by PlasFlow based on draft Nanopore sequencing. Significance was determined using a Mann Whitney U Test (****P<.0001).

**Table S1. Metadata for all Saskatchewan poultry E. coli isolates analyzed in this study.**

| | | | | | | | Polystyrene Biofilm Testing | | | | | |
|---|---|---|---|---|---|---|---|---|---|---|---|---|
| **Sample Name** | **Outbreak #** | **Disease or Healthy** | **Organ** | **Curli** | **Cellulose** | **Phylogroup** | **BHI** | **1/2 TSB** | **M63** | **Disinfectant Testing** | **# Nanopore Reads** | **AMR profile** |
| **23315-H1** | 1 | Disease | Heart | + | - | C | xx | | | | 16,000 | Tet |
| **23315-L3** | 1 | Disease | Liver | + | - | C | | | | | 80,000 | Tet |
| **23315-S2** | 1 | Disease | Spleen | + | - | A | xx | | | | 128,000 | Tet |
| **23315-C4** | 1 | Healthy | Cecum | + | + | A | xx | | | | 140,000 | Amp Sulbactam/Ampicillin/Aztreonam/Cefazolin/Cefepime/Cefotaxim/Ceftriaxone/Cefuroxime/Tetracycline |
| **23315-C5** | 1 | Healthy | Cecum | + | + | G | | | | | 12,000 | Amp Sulbactam/Ampicillin/Aztreonam/Cefazolin/Cefepime/Cefotaxim/Ceftriaxone/Cefuroxime/Tetracycline |
| **23315-C8** | 1 | Healthy | Cecum | - | + | G | | | | | 516,000 | Amp Sulbactam/Ampicillin/Aztreonam/Cefazolin/Cefepime/Cefotaxim/Ceftriaxone/Cefuroxime/Tetracycline |
| **23315-C9** | 1 | Healthy | Cecum | - | - | G | | | | | 88,000 | Susceptible to all |
| | | | | | | | | | | | | |
| **3862-H1** | 2 | Disease | Heart | + | + | E | xx | | | | 123,054 | Susceptible to all |
| **3862-L1** | 2 | Disease | Liver | + | + | E | | | | | 176,000 | Susceptible to all |
| **3862-S1** | 2 | Disease | Spleen | + | + | B1 | xx | | | | 80,000 | Susceptible to all |
| **3862-S2** | 2 | Disease | Spleen | + | + | A | xx | | | | 516,000 | Aztreonam |
| **3862-C2** | 2 | Healthy | Cecum | - | - | A | | | | | 480,000 | Susceptible to all |
| **3862-C6** | 2 | Healthy | Cecum | + | + | G | xx | | | | 356,000 | Susceptible to all |
| **3862-C8** | 2 | Healthy | Cecum | + | - | F | | | | | 112,000 | Susceptible to all |
| **3862-C9** | 2 | Healthy | Cecum | + | - | F | | | | | 64,000 | Tet |
| **3862-C11** | 2 | Healthy | Cecum | - | - | E | | | | | 32,000 | Susceptible to all |
| | | | | | | | | | | | | |
| **9619-1H2** | 3 | Disease | Heart | + | + | G | xx | | | xx | 68,000 | Int-Amp Sulbactam/Ampicillin/Tet/Trimet&Sulfa |
| **9619-2L1** | 3 | Disease | Liver | + | + | G | | | | | 120,000 | Amox K Clav/Amp Sulbactam/Ampicillin/Aztreonam/Cefazolin/Cefotaxime/Cefoxitin/Ceftazidime/ceftriaxone/Cefuroxime/Gentamicin/Tetracycline/Tobramycin |
| **9619-2S1** | 3 | Disease | Spleen | + | + | B1 | | | | | 96,000 | Susceptible to all |
| **9619-3L1** | 3 | Disease | Liver | - | - | A | | | xx | xx | 88,000 | Susceptible to all |
| **9619-3S1** | 3 | Disease | Spleen | + | + | A | | | | | 152,000 | Gentamicin/Tet/Tobramycin |
| **9619-C1** | 3 | Healthy | Cecum | + | - | A | | | | | 156,000 | Susceptible to all |
| **9619-C6** | 3 | Healthy | Cecum | + | + | B1 | | | | | 136,000 | Susceptible to all |
| **9619-C8** | 3 | Healthy | Cecum | + | + | A | | | | | 72,000 | Susceptible to all |
| | | | | | | | | | | | | |
| **0012-1H1** | 4 | Disease | Heart | + | - | G | | | | | 104,000 | Susceptible to all |
| **0012-2L1** | 4 | Disease | Liver | - | + | B2 | | | | | 192,000 | I-amp sulbactram/ampicillin/Tet/Timet&sulfa |
| **0012-3S1** | 4 | Disease | Spleen | + | - | G | | | | | 116,000 | Susceptible to all |
| **0012-C1** | 4 | Healthy | Cecum | + | - | A | | | xx | xx | 192,000 | Susceptible to all |
| **0012-C5** | 4 | Healthy | Cecum | + | - | A | | | xx | | 168,000 | Susceptible to all |
| **0012-C7** | 4 | Healthy | Cecum | + | + | B1 | | | | | 220,000 | Susceptible to all |
| | | | | | | | | | | | | |
| **9226-1L2** | 5 | Disease | Liver | - | + | D | | | | | 208,000 | Amp Sulbacam/Ampicillin/Gentamicin/Tobramycin |
| **9226-1S1** | 5 | Disease | Spleen | + | + | C | | | | | 76,000 | Tet |
| **9226-2H2** | 5 | Disease | Heart | + | + | D | | xx | | | 52,000 | Amox Kclav/ Amp Sulbactam/Ampicillin/Aztreonam/Cefazolin/Cefotaxime/Cefoxitin/Ceftazidime/Ceftriaxone/Cefuroxime/Gentamicin/Tobramycin |
| **9226-2L1** | 5 | Disease | Liver | + | + | D | | xx | | | 104,000 | I- Amp Sulbactam/Ampicillin/Gentamicin/I-Tobramycin |
| **9226-2S1** | 5 | Disease | Spleen | + | + | D | | xx | | | 64,000 | Amox Kclav/Amp Sulbactam/Ampicillin/Aztreonam/Cefazolin/Cefotaxime/Cefoxitin/Ceftazidime/Ceftriaxone/Cefuroxime/Gentamicin/Tet/Tobramycin |
| **9226-3H1** | 5 | Disease | Heart | + | + | D | | xx | | xx | 52,000 | Amox Kclav/Amp Sulbactam/Ampicillin/Aztreonam/Cefazolin/Cefotaxime/Cefoxitin/Ceftazidime/Ceftriaxone/Cefuroxime/Gentamicin/Tobramycin |
| **9226-3L1** | 5 | Disease | Liver | + | + | D | | xx | | | 64,000 | Amox Kclav/Amp Sulbactam/Ampicillin/Aztreonam/Cefazolin/Cefotaxime/Cefoxitin/Ceftazidime/Ceftriaxone/Cefuroxime/Gentamicin/Tobramycin |
| **9226-C1** | 5 | Healthy | Cecum | - | + | D | | | | | 140,000 | Susceptible to all |
| **9226-C2** | 5 | Healthy | Cecum | - | + | D | | | | | 188,000 | Susceptible to all |
| **9226-C5** | 5 | Healthy | Cecum | - | + | B1 | | | | | 96,000 | I- Amox Kclav/Amp Sulbactam/Ampicillin/I - Pip Tazo |
| | | | | | | | | | | | | |
| **6245-1H1** | 6 | Disease | Heart | + | + | C | xx | | | | 44,000 | Amp Sulbacam/Ampicillin/Gentamicin/Tobramycin |
| **6245-1L1** | 6 | Disease | Liver | + | + | C | xx | | | | 240,000 | Amp Sulbacam/Ampicillin/Gentamicin/Tobramycin |
| **6245-1S2** | 6 | Disease | Spleen | + | + | C | xx | | | | 16,000 | Tet |
| **6245-2H3** | 6 | Disease | Heart | + | + | C | xx | | | xx | 68,000 | I - Amp Sulbatam/Amadpicillin/Gentamicin/Tobramycin |
| **6245-2L1** | 6 | Disease | Liver | + | + | E | xx | | | | 48,000 | Trimeth/Sulfa |
| **6245-3H2** | 6 | Disease | Heart | + | - | G | xx | | | | 36,000 | Tet |
| **6245-3L1** | 6 | Disease | Liver | + | + | G | xx | | | | 40,000 | Tet |
| **6245-3S1** | 6 | Disease | Spleen | + | + | G | xx | | | | 100,000 | Tet |
| **6245-C1** | 6 | Healthy | Cecum | + | + | A | xx | xx | | | 48,000 | Susceptible to all |
| **6245-C2** | 6 | Healthy | Cecum | + | + | A | xx | xx | | xx | 132,000 | Susceptible to all |
| **6245-C5** | 6 | Healthy | Cecum | + | + | A | xx | xx | | xx | 68,000 | Susceptible to all |
| **6245-C6** | 6 | Healthy | Cecum | + | + | A | xx | | | | 32,000 | Gentamicin/I - Tobramycin/Nitrofurantoin |
| **6245-C4** | 6 | Healthy | Cecum | + | + | D | xx | | | | 60,000 | Tet |

**E. coli strains that were recovered from polystyrene pegs at greater than $10^7$ colony forming units per mL**

**Table S1. Continued**

| Sample Name | Outbreak # | Disease or Healthy | Organ | Curli | Cellulose | Phylogroup | Polystyrene Biofilm Testing: BHI | Polystyrene Biofilm Testing: 1/2 TSB | Polystyrene Biofilm Testing: M63 | Disinfectant Testing | # Nanopore Reads | AMR profile |
|---|---|---|---|---|---|---|---|---|---|---|---|---|
| **9413-1H1** | 7 | Disease | Heart | + | + | A | | | | | 132,000 | I- Amp Sulbactam/Ampicillin/Getamicin/Tobramycin |
| **9413-1S2** | 7 | Disease | Spleen | + | + | G | | | xx | xx | 68,000 | I- Amp Sulbactam/Ampicillin/Getamicin/Tobramycin |
| **9413-2H1** | 7 | Disease | Heart | + | + | A | | | | | 132,000 | Gentamicin/Tet |
| **9413-2S2** | 7 | Disease | Spleen | + | + | A | | | xx | | 60,000 | Tet |
| **9413-3S1** | 7 | Disease | Spleen | + | + | B2 | | | | | 152,000 | Susceptible to all |
| **9413-C1** | 7 | Healthy | Cecum | - | - | G | | | | | 112,000 | Susceptible to all |
| **9413-C4** | 7 | Healthy | Cecum | + | + | B1 | | | | | 84,000 | Aztreonam |
| | | | | | | | | | | | | |
| **6041-1H1** | 8 | Disease | Heart | + | + | D | | | | | 88,000 | Trimeth/Sulfa |
| **6041-1L2** | 8 | Disease | Liver | + | + | D | | | | | 192,000 | Trimeth/Sulfa |
| **6041-2L1** | 8 | Disease | Liver | + | + | D | | | | | 124,000 | I- Amp Sulbactam/Ampicillin/Gentamicin/Tobramycin |
| **6041-3L1** | 8 | Disease | Liver | + | - | G | xx | | xx | xx | 28,000 | Amox Kclav/Amp Sulbactam/Ampicillin/Aztreonam/Cefazolin/Cefotaxime/Cefoxitin/Ceftazidime/Ceftriaxone/Cefuroxime/Gentamicin/Tobramycin |
| **6041-3S1** | 8 | Disease | Spleen | + | - | G | xx | xx | | | 92,000 | Gentamicin/I-tobramycin |
| **6041-C2** | 8 | Healthy | Cecum | + | + | A | | | xx | xx | 60,000 | Susceptible to all |
| **6041-C6** | 8 | Healthy | Cecum | + | - | A | | | xx | | 88,000 | Susceptible to all |
| **6041-C9** | 8 | Healthy | Cecum | + | + | A | | | xx | | 84,000 | Susceptible to all |
| | | | | | | | | | | | | |
| **0205-1L3** | 9 | Disease | Liver | - | + | G | | | | | 84,000 | Susceptible to all |
| **0205-2L2** | 9 | Disease | Liver | - | + | G | | | | | 220,000 | Susceptible to all |
| **0205-3H1** | 9 | Disease | Heart | + | + | D | xx | xx | | | 164,000 | Amp Sulbacam/Ampicillin/Tetracycline/Trimeth/Sulfa |
| **0205-3S1** | 9 | Disease | Spleen | + | + | G | xx | xx | | | 104,000 | I- Amox Kclav/Amp Sulbactam/Ampicillin/Tetracycline/Trimeth/Sulfa |
| **0205-C3** | 9 | Healthy | Cecum | + | - | A | xx | | | | 232,000 | Susceptible to all |
| **0205-C7** | 9 | Healthy | Cecum | - | - | E | xx | | | | 184,000 | Susceptible to all |
| **0205-C9** | 9 | Healthy | Cecum | + | + | A | xx | xx | | | 192,000 | Susceptible to all |
| | | | | | | | | | | | | |
| **2402-1H1** | 10 | Disease | Heart | + | + | E | | | | | 176,000 | Susceptible to all |
| **2402-2L1** | 10 | Disease | Liver | + | + | G | | | | | 180,000 | Susceptible to all |
| **2402-3S1** | 10 | Disease | Spleen | + | + | G | | | | | 84,000 | Susceptible to all |
| **2402-C2** | 10 | Healthy | Cecum | + | + | A | | | | | 88,000 | Gentamicin/Tetracycline/Tobramycin |
| **2402-C3** | 10 | Healthy | Cecum | + | - | B1 | | xx | | | 92,000 | Gentamicin/Tetracycline/Tobramycin |
| **2402-C4** | 10 | Healthy | Cecum | + | + | A | | | | | 104,000 | Gentamicin/Tetracycline/Tobramycin |
| | | | | | | | | | | | | |
| **4957-1H1** | 11 | Disease | Heart | + | + | A | xx | | | | 85,000 | Susceptible to all |
| **4957-1H2** | 11 | Disease | Heart | - | - | B2 | xx | | | | 124,000 | Nitrofurantoin |
| **4957-2L1** | 11 | Disease | Liver | + | + | A | xx | | | | 104,000 | I-Amp sulbactram/ampicillin/Gentimicin/Tobramycin |
| **4957-2L3** | 11 | Disease | Liver | - | + | B2 | xx | xx | | xx | 64,000 | Susceptible to all |
| **4957-3S1** | 11 | Disease | Spleen | + | - | A | xx | | | | 332,000 | I- Amp Sulbactam/Ampicillin/Gentamycin/Tobramycin/Tet |
| **4957-3S3** | 11 | Disease | Spleen | + | + | A | xx | | | | 132,000 | I- Amp Sulbactam/Ampicillin/Gentamicin/Tetracycline/Tobramycin |
| **4957-C1** | 11 | Healthy | Cecum | + | - | A | xx | xx | | | 124,000 | Susceptible to all |
| **4957-C3** | 11 | Healthy | Cecum | + | + | A | xx | | | | 84,000 | Susceptible to all |
| **4957-C5** | 11 | Healthy | Cecum | + | + | A | xx | | | xx | 96,000 | Susceptible to all |
| **4957-C6** | 11 | Healthy | Cecum | + | + | A | xx | | | | 164,000 | Susceptible to all |
| | | | | | | | | | | | | |
| **7578-1H1** | 12 | Disease | Heart | + | + | B1 | xx | | | | 104,000 | Susceptible to all |
| **7578-2H2** | 12 | Disease | Heart | + | + | A | xx | | | | 20,000 | I- Amp Sulbactam/Ampicillin/Gentamicin/Tobramycin |
| **7578-2H3** | 12 | Disease | Heart | + | + | A | xx | | | | 96,000 | I- Amp Sulbactam/Ampicillin/Gentamicin/Tobramycin |

(yellow) **E. coli strains that were recovered from polystyrene pegs at greater than $10^7$ colony forming units per mL**

**Table S2** Summary of disinfectants and their concentrations used in the study

| **Disinfectants** | **Virocid** | **Virkon™** | **(DDAC)** | **$H_2O_2$** |
|---|---|---|---|---|
| Manufacturer/Supplier | CID LINES N.V., Ieper, Belgium. | Antec International LTD., Suffolk, UK. | KleenGlow, PACE Chemicals LTD., BC, Canada. | Plant Life Products, BC, Canada. |
| Group of Ingredients | Two different quaternary ammonia, modern aldehyde, alcohol, pineoil and other ingredients | Pentapotassium bis(peroxymonosulphate) bis(sulphate), Sodium Dodecylbenzene Sulfonate, Butanedioic acid, 2-hydroxy-sulphamic acid, Potassium hydrogen sulphate, Sodium chloride, Dipotassium peroxodisulphate, Dipotassium disulphate, Dipentene. | Isopropyl Alcohol, Didecyldimethyl Ammonium Chloride (DDAC) and Ethanol | $H_2O_2$ and Water |
| Active Ingredients | Alkyl dimethyl benzyl ammonium chloride (17.06%); Didecyl dimethyl ammonium chloride (7.8%); Glutaraldehyde (10.8%) and other ingredients (64.415%). | Potassium monopersulfate (21.4%) | DDAC (7.5%) | $H_2O_2$ (29%) technical grade |
| pH | 6.5 | 5.0 – 6.5 | 6.0 – 7.0 | 4.7 |
| Recommended Conc. | 0.25% | 1% | 300 ppm | Not Available |
| Recommended contact time | 10-15 min | 10 min | Not Available | Not Available |
| **Concentrations and exposure time used in this study** | | | | |
| Concentration used on Planktonic cells for 18 h[#] | 0.0125% – 0.000025% | 1% – 0.002% | 30ppm – 0.06ppm | 0.5% – 0.001% |
| Concentration used on Planktonic or biofilm cells for 30 min | 0.25% – 0.0005% | 2% – 0.004% | 300ppm – 0.6ppm | 4% – 0.008% |

**Table S3.** Determination of MIC and MBC for four commercial disinfectants against *E. coli* isolates from Saskatchewan poultry.

| S.No | Strain ID | Virocid (%) | | Virkon™ (%) | | DDAC (ppm) | | $H_2O_2$ (%) | |
|---|---|---|---|---|---|---|---|---|---|
| | | MIC# | MBC# | MIC# | MBC# | MIC# | MBC# | MIC# | MBC# |
| **Systemic *E.coli* isolates** | | | | | | | | | |
| 1 | 9226-3H1 | 0.003 | 0.003 | 0.25 | 0.25 | 3.75 | 7.5 | 0.063 | 0.125 |
| 2 | 4957-2L3 | 0.002 | 0.003 | 0.13 | 0.25 | 3.75 | 3.75 | 0.03 | 0.03 |
| 3 | 6245-2H3 | 0.003 | 0.006 | 0.25 | 0.25 | 1.88 | 3.75 | 0.063 | 0.063 |
| 4 | 9619-1H2 | 0.003 | 0.006 | 0.25 | 0.25 | 3.75 | 3.75 | 0.063 | 0.063 |
| 5 | 6041-3L1 | 0.003 | 0.006 | 0.25 | 0.25 | 3.75 | 7.5 | 0.03 | 0.03 |
| 6 | 9619-3L1 | 0.003 | 0.003 | 0.25 | 0.25 | 3.75 | 3.75 | 0.063 | 0.063 |
| 7 | 9413-1S2 | 0.003 | 0.003 | 0.25 | 0.25 | 3.75 | 7.5 | 0.031 | 0.063 |
| **Cecal *E.coli* isolates** | | | | | | | | | |
| 8 | 6245-C2 | 0.003 | 0.003 | 0.25 | 0.25 | 3.75 | 3.75 | 0.063 | 0.063 |
| 9 | 4957-C5 | 0.003 | 0.003 | 0.25 | 0.25 | 3.75 | 3.75 | 0.063 | 0.063 |
| 10 | 6245-C5 | 0.003 | 0.003 | 0.25 | 0.25 | 3.75 | 3.75 | 0.063 | 0.063 |
| 11 | 0012-C1 | 0.003 | 0.003 | 0.25 | 0.25 | 3.75 | 3.75 | 0.03 | 0.063 |
| 12 | 6041-C2 | 0.003 | 0.003 | 0.25 | 0.25 | 3.75 | 3.75 | 0.063 | 0.125 |

# MIC and MBC of disinfectants for each *E. coli* strain were determined following CLSI guidelines (CLSI, 2020).

**Table S4.** The mean $log_{10}$ density (CFU) of starting inoculum from planktonic and biofilm cells before disinfectant challenge.

| **S.No** | **Strain ID** | **Starting cells (Mean log 10 ±SD)** | |
|---|---|---|---|
| | | **Planktonic cells** | **Biofilm cells** |
| 1 | 9226-3H1 | 7.68 ± 0.35 | 8.49 ± 0.63 |
| 2 | 4957-2L3 | 7.57 ± 0.65 | 6.84 ± 0.21 |
| 3 | 6245-2H3 | 7.57 ± 0.31 | 7.90 ± 0.55 |
| 4 | 9619-1H2 | 7.45 ± 0.30 | 6.86 ± 0.05 |
| 5 | 6041-3L1 | 7.48 ± 0.29 | 8.18 ± 0.46 |
| 6 | 9619-3L1 | 7.45 ± 0.41 | 8.47 ± 0.32 |
| 7 | 9413-1S2 | 7.24 ± 0.54 | 7.11 ± 0.29 |
| 8 | 6245-C2 | 7.33 ± 0.42 | 8.04 ± 0.45 |
| 9 | 4957-C5 | 7.32 ± 0.41 | 7.07 ± 0.37 |
| 10 | 6245-C5 | 7.56 ± 0.36 | 7.85 ± 0.54 |
| 11 | 0012-C1 | 7.21 ± 0.17 | 7.86 ± 0.59 |
| 12 | 6041-C2 | 7.45 ± 0.30 | 8.22 ± 0.08 |
